# Comprehensive analysis identifies novel targets of gemcitabine to improve chemotherapy treatment strategies for colorectal cancer

**DOI:** 10.3389/fendo.2023.1170526

**Published:** 2023-08-17

**Authors:** Xinxin Zeng, Liyue Sun, Xiaomei Ling, Yuying Jiang, Ju Shen, Lei Liang, Xuhui Zhang

**Affiliations:** ^1^ Second Department of Oncology, The Affiliated Guangdong Second Provincial General Hospital of Jinan University, Guangzhou, China; ^2^ Medical Research Center, The Affiliated Guangdong Second Provincial General Hospital of Jinan University, Guangzhou, China; ^3^ Department of Radiation Oncology, Guangdong Medical University, Zhanjiang, China; ^4^ Guangdong Engineering Research Center of Chinese Medicine & Disease Susceptibility, School of Traditional Chinese Medicine, Jinan University, Guangzhou, China

**Keywords:** colorectal cancer, chemotherapy, gemcitabine, combination drug, CALB2, GPX3

## Abstract

**Background:**

Gemcitabine (GEM) is a second-line anticancer drug of choice for some colorectal cancer (CRC) patients, and GEM inability to be commonly available in the clinic due to the lack of clarity of the exact action targets.

**Methods:**

The half maximal inhibitory concentration (IC50) of GEM treatment for 42 CRC cell lines were accessed from the Genomics of Drug sensitivity in Cancer (GDSC) database. High-throughput sequencing data of CRC patients were captured in The Cancer Genome Atlas (TCGA) and Weighted correlation network analysis (WGCNA) was conducted. Pearson correlations were derived for GEM potency-related genes. Differential analysis was conducted in the TCGA cohort to obtain CRC development-related genes (CDRGs), and univariate COX model analysis was performed on CDRGs overlapping with GEM potency-related genes to obtain CDRGs affecting CRC prognosis. Hub genes affecting GEM potency were identified by Spearman correlation.

**Results:**

CALB2 and GPX3 were identified as potential targets for GEM treatment of CRC *via* prognostic analysis, which we also observed to be elevated with elevated clinical stage in CRC patients. The enhanced expression of CALB2 and GPX3 genes identified in the pathway analysis might inhibit the body metabolism as well as activate immune and inflammation related pathways. In addition, we found that CALB2 and GPX3 could also be considered as prognostic biomarkers in pan-cancer. Finally, we found that CALB2 and GPX3 were remarkably associated with the drug sensitivity of MG-132, Dasatinib, Shikonin, Midostaurin, MS-275, and Z-LNle-CHO, which were expected to be the drugs of choice for GEM combination.

**Conclusion:**

CALB2 and GPX3 represent prognostic biomarkers for CRC and they might be potential action targets for GEM. Our study offered innovative ideas for GEM administration strategies.

## Introduction

Genetic alterations resulting from somatic mutations or gene fusions contributed to colorectal cancer (CRC) being a highly heterogeneous cancer due to the coexistence of multiple pathogenic mechanisms (1). Over million people developed CRC and hundreds of thousands of CRC patients died yearly (2). The reason for the high mortality rate of CRC was that most patients already had metastases when diagnosed (2). Currently, surgical resection was the dominant treatment option for CRC, and chemotherapy was generally considered for patients with local metastases, but tumor heterogeneity caused some CRC patients to develop chemotherapy resistance (3). Identifying effective treatment modalities is crucial to improve survival in CRC.

Gemcitabine (GEM) was second-line resistant drug, and high resistance limited the applicability of GEM in the clinic (4). GEM could hardly be treated as first-line chemotherapeutic agent due to enzymatic deamination, low clearance and high resistance (4). Recent report by Chocry et al. (5) illustrated that GEM was a potential alternative drug when CRC patients developed Oxaliplatin resistance. GEM remained an alternative option for CRC patients. In recent years, several studies have focused on the administration of GEM to tumor cells using nanotechnology to enhance the efficacy of GEM (6). It was evident that current drug delivery strategies and the absence of an exact drug target were the major limiting factors for GEM. Studies suggested that novel drug delivery modalities could assist GEM as cancer-targeted drugs, but related studies were still exploring (7). However, studies focusing on marker genes identification from GEM-related genes in CRC remain limited.

RNA sequencing (RNA-Seq) is a high-throughput sequencing technology used to study transcriptomes, enabling more accurate quantification of gene expression levels, both to identify novel transcript sequences and for differential expression studies (8). In this study, based on multiple databases, we investigated the hub genes of GEM acting in CRC. we comprehensively explored the association between hub genes and multiple cancer prognosis, tumor-infiltrating immune cells (TIIC) in the tumor microenvironment (TME), and chemotherapeutic drug sensitivity. Our study explored the potential of GEM as CRC-targeting agent and the potential contribution of these hub genes in CRC prognosis.

## Materials and methods

### Data acquisition and pre-processing

Transcriptome high-throughput sequencing datasets of CRC patients as well as normal tissues and corresponding clinical phenotype data were obtained from The Cancer Genome Atlas (TCGA, https://portal.gdc.cancer.gov/) website. The gene expression was showed as log2(TPM+1). Tumor simples whose survival time was absence and less than 0 days of survival were removed, and 432 tumor samples as well as 41 normal tissue samples were retained. The microarray sequencing datasets GSE17536, GSE17537, GSE17538, GSE39582 of CRC patients were loaded from the GENE EXPRESSION OMNIBUS (GEO, https://www.ncbi.nlm.nih.gov/geo/) website. Normal tissue samples, samples with missing clinical follow-up information, and survival time less than 0 days were excluded in 4 cohorts. 177, 55, 232, and 573 tumor samples were retained in GSE17536, GSE17537, GSE17538, and GSE39582, respectively, for follow-up studies. The clinical information was showed in **Table 1**. The half maximal inhibitory concentration (IC50) data for 42 CRC cell lines treated with GEM were accessed from the Genomics of Drug sensitivity in Cancer database (GDSC, https://www.cancerrxgene.org/).

**Table 1:** The clinical information of TCGA dataset.

TCGA		
Gender		
	male	232
	female	200
T stage		
	T1	11
	T2	75
	T3	295
	T4	50
	Unknown	1
N stage		
	N0	253
	N1	102
	N2	77
M stage		
	M0	319
	M1	61
	Unknown	52
Stage		
	I	73
	II	164
	III	123
	IV	61
	Unknown	11
Status		
	Alive	339
	Dead	93
Age	<=65	183
	>65	249

### WGCNA Analysis

In this study, Weighted correlation network analysis (WGCNA) was performed on genes in the TCGA dataset by referring to the method of Langfelder et al. (9) using WGCNA R package (9). The parameters were set: correlation coefficient > 0.85, minimum number of module genes > 50. After merging similar gene modules, principal component analysis (PCA) was performed on the final gene modules, the first principal component of each module was analyzed as Module eigengene E with IC50 values of GEM for pearson correlation analysis to determine the gene modules affecting GEM potency, and the GEM potency-related genes within the modules were included for subsequent analysis.

### Identification of CRC development-related genes

In the TCGA cohort, differential analysis was performed using the limma package (10) to identify CRC development-related genes (CDRGs) in tumor tissues using normal tissues as controls. CDRGs were intersected with GEM potency-related genes to obtain candidate CDRGs affecting GEM potency. univariate COX model analysis based on the expression matrix of these CDRGs and the survival information of CRC patients in the TCGA cohort was performed to identify candidate hub genes associated with CRC survival. Finally, based on the expression levels of candidate hub genes, Spearman correlation between them and IC50 values of GEM was assessed to determine the hub genes of GEM for CRC treatment.

### Prognostic impact of hub genes on CRC

In the TCGA, GSE17536, GSE17537, GSE17538, and GSE39582 cohorts, CRC patients were clustered into high and low expression groups using the survminer code package (https://rpkgs.datanovia.com/survminer/index.html) to determine the optimal group cut-off values, and Kaplan-Meier (K-M) survival curves were plotted for patients in the high- and low-expression groups using survminer R package (11).

### Association between hub genes and CRC clinical phenotypes

In the TCGA cohort, the expression levels of hub genes were compared among patients in the Stage, TNM. Stage subgroups to explore the association between hub genes and CRC clinical phenotypes.

### Gene set variation analysis

In the TCGA dataset, we performed Gene Set Variation Analysis (GSVA) using the GSVA code package (12) to resolve CALB2 and GPX3 regulated pathways. To calculate potential connections between CALB2 and GPX3 and their regulatory pathways, we performed spearman correlation analysis between CALB2 and GPX3 expression levels and GSVA scores of the pathways to mine the pathways markedly associated with CALB2 and GPX3.

### Connection between CALB2 and GPX3 and TIIC

In the TCGA dataset, we used the Estimation of Stromal and Immune cells in Malignant Tumours using Expression data (ESTIMATE) algorithm (13) to assess the TIICs in TME of CRC patients with ImmuneScore, StromalScore of stromal cells, and ESTIMATEScore. the CIBERSORT algorithm (14) was utilized to assess the relative infiltration scores of 22 TIICs in TME and to calculate the spearman correlation between CALB2 and GPX3 and TIICs. Further, 28 signatures in pan-cancer that could predict Checkpoint Blockade response were captured from the research of Charoentong et al. (15) and ssGSEA Score was calculated (16). Finally, the correlation between CALB2 and GPX3 and the 28 signatures capable of predicting Checkpoint Blockade response was assessed before using the mantel test and pearson correlation.

### Prognostic utility of CALB2 and GPX3 in pan-cancer

The expression profiles of 32 cancers were downloaded from Sangerbox (http://vip.sangerbox.com) (17) and the expression levels of CALB2 and GPX3 in tumor tissues and normal tissues were assessed using the wilcox test. The survival time and survival status of patients with 32 cancers in TCGA were extracted from the study of Liu et al. (18), and the prognostic role of CALB2 and GPX3 was assessed by plotting K-M survival curves for the groups using the optimal group cut-off values obtained from the survminer code package.

### Pharmaceutical sensitivity analysis of CALB2 and GPX3

In the TCGA cohort, we utilized the pRRophetic code package (19) to predict the IC50 for 51 chemotherapeutic agents in the high- and low-expression groups of CRC patients with CALB2 and GPX3. p-values of the IC50 for the drugs were examined by wilcox.test and histograms were plotted. We screened the spearman correlation between the three groups of drugs with the largest and smallest IC50 and CALB2 and GPX3.

### Statistical analysis

All statistical analyses were performed by R software (version 3.62). Wilcoxon nonparametric rank sum test was used to analyze the differences, and a P-value < 0.05 was considered significant unless otherwise specified.

## Results

### Identification of GEM potency-related genes

**Figure 1:**
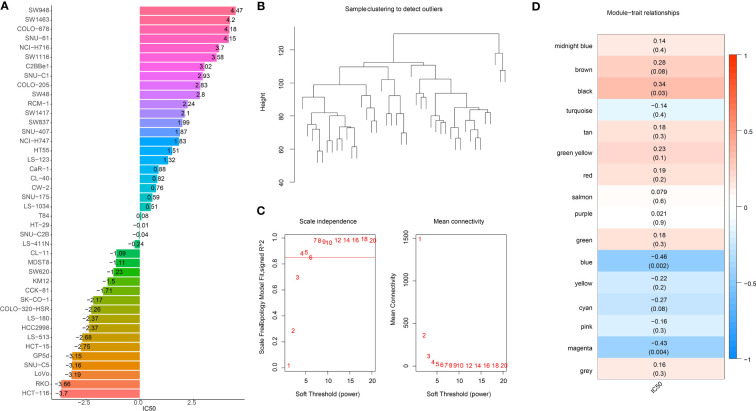
Identification of GEM potency-related genes. **(A)** IC50 of Gemcitabine-treated CRC cells **(B)** Clustering tree of TCGA samples **(C)** Scale-free network analysis **(D)** Heatmap of IC50 of Gemcitabine with pearson correlation of Module eigengene E of gene module.

The workflow was showed in **Figure S1**. Firstly, we extracted the IC50 data of 42 CRC cell lines in response to GEM from the GDSC2 database (**Figure 1A**). Based on the dynamic shear tree algorithm, 16 gene modules were identified *via* WGCNA analysis by selecting a soft threshold β=4 to construct a scale-free network (**Figures 1B, C**). The GEM drug IC50 data of 42 CRC cells were considered as clinical data, and Pearson correlation analysis was performed with the first principal component Module eigengene E of the 16 gene modules to select the most relevant gene modules for GEM efficacy. We found that genes within the blue and magenta modules were remarkably negatively correlated with GEM efficacy (**Figure 1D**), and this result suggested that genes within these two modules might be potential target genes for GEM treatment of CRC. Therefore, blue and magenta intramodule genes were selected for subsequent study.

### Hub genes influencing GEM potency

**Figure 2:**
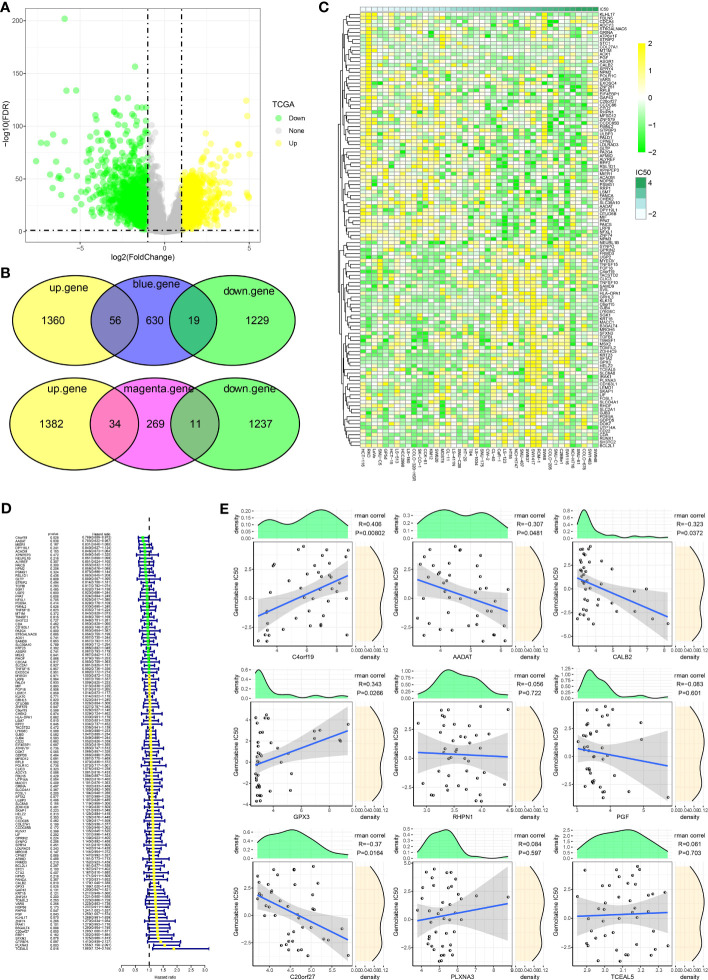
Hub genes influencing GEM potency. **(A)** Volcano map of CDRGs **(B)** Venn diagram of potential hub genes influencing the potency of GEM **(C)** Heatmap of 120-CDRGs expression **(D)** Forest plot of univariate COX model for 120-CDRGs **(E)** Correlation analysis between CDRGs and IC50 values of Gemcitabine.

To further identify hub genes for GEM potency, we identified CDRGs in the TCGA dataset. 2664 CDRGs were identified by differential analysis, including 1416 up-regulated CDRGs and 1248 down-regulated CDRGs (**Figure 2A**). Subsequently, candidate CDRGs affecting GEM potency were identified by Venn diagram analysis. we intersected the CDRGs in TCGA with genes within the blue and magenta modules, respectively. there were 56 up-regulated basal CDRGs and 19 down-regulated CDRGs in the blue module, and 34 up-regulated CDRGs and 11 down-regulated CDRGs in the magenta module (**Figure 2B**). These 120 CDRGs may be potential hub genes affecting the potency of GEM. we demonstrated the expression levels of 120-CDRGs in 42 CRC cells by heat map (**Figure 2C**). 9 CDRGs associated with CRC prognosis was identified by univariate COX regression model (**Figure 2D**). Finally, Spearman correlation analysis based on the expression levels of the 9-CDRGs with the IC50 values of GEM was conducted. We identified C4orf19, GPX3, C20orf27, AADAT and CALB2 as hub genes affecting the potency of GEM, with C4orf19, GPX3 and C20orf27 showing remarkable positive correlation with IC50 of GEM, and AADAT and CALB2 showing remarkable negative correlation with IC50 of GEM (**Figure 2E**). Overall, these results suggest that C4orf19, GPX3, C20orf27, AADAT, and CALB2 might be the candidate hub genes for GEM treatment of CRC.

### Correlation of 5-hub genes with CRC prognosis and clinical information

**Figure 3:**
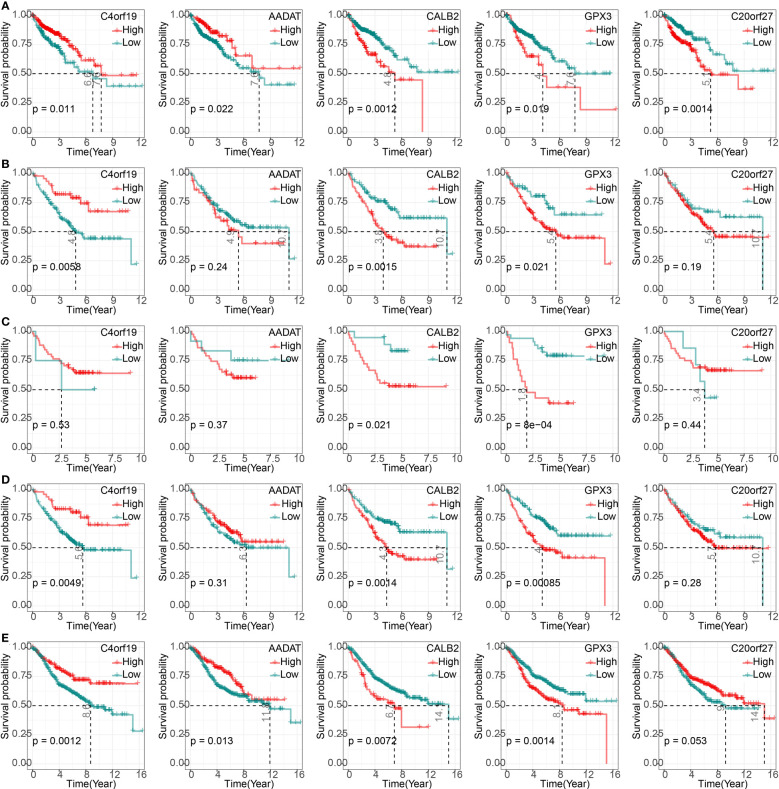
Correlation of 5-hub genes with CRC prognosis. **(A–E)** K-M survival curves of 5-hub genes in TCGA, GSE17536, GSE17537, GSE17538, and GSE39582 cohorts.

**Figure 4:**
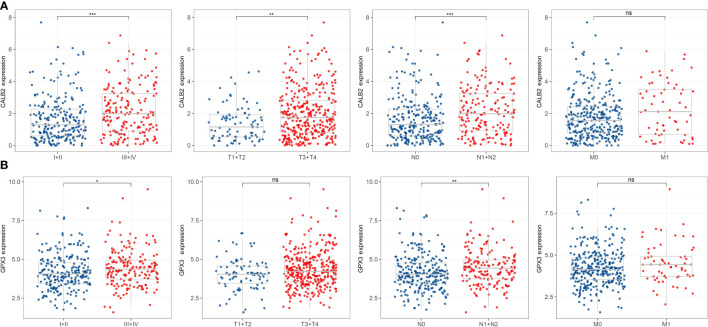
Correlation of 5-hub genes with CRC clinical information. ns, p>0.05, *p<0.05, **p<0.01, ***p<0.001. **(A, B)** Expression levels of CALB2 and GPX3 in clinical subgroups.

We found that high expression of C4orf19 and AADAT resulted the promising prognosis of CRC patients, and low expression of GPX3, C20orf27 and CALB2 resulted to better prognosis of CRC patients (**Figure 3A**). Subsequently, we further validated the relationship between 5-hub genes and CRC prognosis in four external GEO datasets (GSE17536, GSE17537, GSE17538, GSE39582). We found that CALB2 and GPX3 showed concordance in the four datasets, and patients in the high expression group had markedly poor prognosis (**Figures 3B–E**, p<0.05). In combination with the TCGA dataset, CALB2 and GPX3 might be hub genes for GEM treatment to CRC. To further investigate the potential association between CALB2 and GPX3 expression and Stage, TNM. Stage, we found that CALB2 and GPX3 expression increased with Stage, T. Stage, and N. Stage staging (**Figure 4A**). The expression of GPX3 increased with Stage, N. Stage (**Figure 4B**). The ridge analysis of CALB2 and GPX3 in 5 dataset was presented in **Figure S1**. those findings indicated that the 5 hub genes were closely associated with development of CRC.

### Biological pathways involved in CALB2 and GPX3

**Figure 5:**
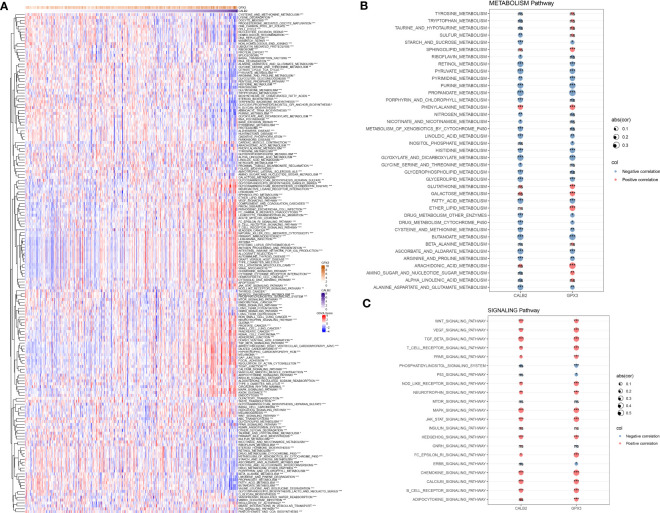
Biological pathways involved in CALB2 and GPX3. **(A)** Heatmap of GSVA results for CALB2 and GPX3 **(B)** Bubble plot of metabolism-related pathway enrichment score correlation analysis with CALB2 and GPX3 expression **(C)** Bubble plot of correlation analysis of signaling pathway enrichment score with CALB2 and GPX3 expression. ns p>0.05, *p<0.05, **p<0.01, ***p<0.001.

To further resolve the pathways potentially regulated by CALB2 and GPX3 in the TCGA dataset, we compared the pathways with significantly enriched pathways in tumor tissues and paraneoplastic tissues by GSVA method and calculated the GSVA scores of all pathways. We found that 172 KEGG pathways were significantly different in tumor tissues and paracancerous tissues, and heatmap was presented to show the GSVA enrichment fractions of 172 differential pathways in tumor tissues (**Figure 5A**). Accumulating studies indicated that tumor development was closely related to metabolic and signaling pathways in the organism (20, 21), we extracted 172 metabolic and signaling-related pathways in the organism among KEGG pathways and calculated their correlations with CALB2 and GPX3. We found that CALB2 was significantly associated with 27 METABOLISM Pathways and 14 SIGNALING Pathways, respectively, and GPX3 was significantly associated with 26 METABOLISM Pathways and 17 SIGNALING Pathways, respectively (**Figures 5B, C**). Those results revealed that the enhanced expression of CALB2 and GPX3 might inhibit the organism metabolism. Among the signaling pathways, it was found that the enhanced expression of CALB2 and GPX3 would activate immune and inflammation-related pathways.

### Correlation between CALB2 and GPX3 and immune microenvironment

**Figure 6:**
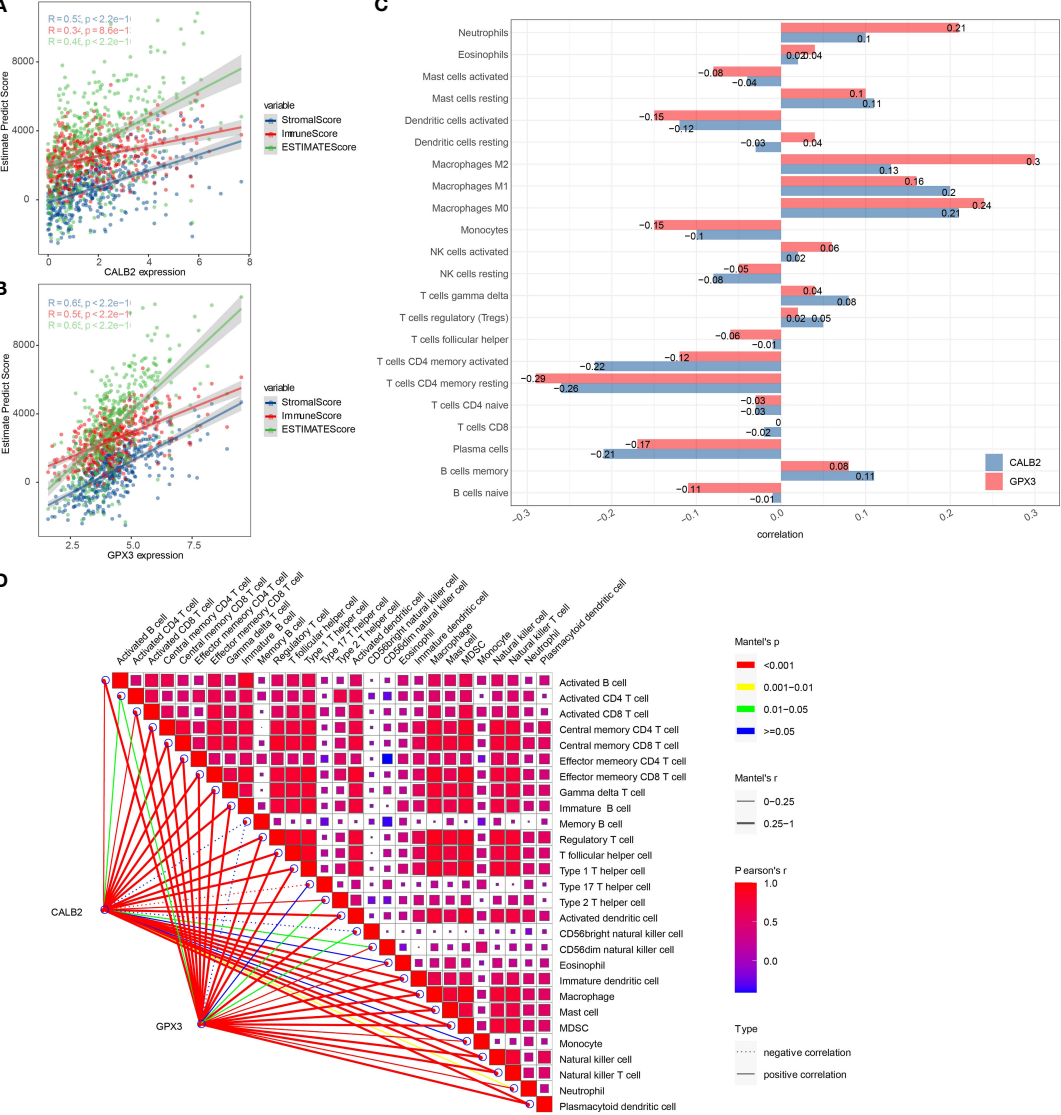
Correlation between CALB2 and GPX3 and immune microenvironment. **(A)** Scatter plot of CALB2 expression correlation with immune and stromal scores **(B)** Scatter plot of GPX3 expression correlation with immune and stromal scores **(C)** Histogram of the correlation between CALB2 and GPX3 expression and 22 TIICs. **(D)** Pearson analysis between 28 immune cell score and CALB2/GPX3.

To investigate the potential connection between CALB2 and GPX3 and immunity, we evaluated the correlation between CALB2 and GPX3 and immune cell infiltration scores in TME. First, we evaluated immune cell scores in TME of CRC patients in the TCGA cohort by ESTIMATE and CIBERSORT algorithms and found correlations between CALB2 and GPX3 expression and immune scores by spearman correlation analysis. We found that the expression levels of CALB2 and GPX3 were significantly correlated with the StromalScore, ImmuneScore, and ESTIMATEScore of CRC (p<0.05) (**Figures 6A, B**). Except for the infiltration score of Dendritic cells resting, the infiltration scores of the remaining 21 immune cells showed concordance with the expression of CALB2 and GPX3 (**Figure 6C**). Finally, we calculated the ssGSEA enrichment scores of 28 signatures that could predict Checkpoint Blockade response. The results of the mantel test and pearson correlation showed that CALB2 and GPX3 expression were significantly correlated with most signatures that could predict the checkpoint Blockade response (**Figure 6D**). Immunocyte analysis implied that CALB2 and GPX3 had obviously correlated 7 immune cells (**Figure S2**). These results indicated that the immune function of CRC patients was enhanced with the increasing expression of CALB2 and GPX3 genes.

### The role of CALB2 and GPX3 in pan-cancer

**Figure 7:**
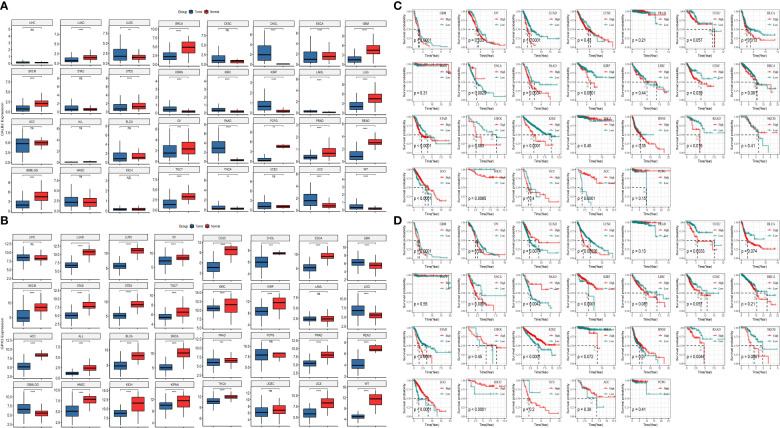
The role of CALB2 and GPX3 in pan-cancer. **(A, B)** Expression of CALB2 and GPX3 in pan-cancer **(C, D)** K-M survival curves of CALB2 and GPX3 in multiple cancer types.

To further analyze the prognostic value of CALB2 and GPX3 in pan-cancer, we compared the expression levels of CALB2 and GPX3 in 32 tumor tissues and paraneoplastic tissues from TCGA and GTEx data. The results showed that CALB2 and GPX3 were highly expressed in most tumor tissues (**Figures 7A, B**). Next, the K-M survival curves demonstrated the prognostic status in the high- and low-expression groups of CALB2 and GPX3 in 26 cancers. We found that high CALB2 expression was associated with poorer prognosis in GBM, OV, LUAD, BLCA, PAAD, KIRP, CESC, STAD, CHOL, KIRC, READ, and KICH (p<0.05) (**Figure 7C**). High CALB2 expression was associated with more favorable prognosis in ESCA, LGG, and ACC patients (p<0.05) (**Figure 7C**). High GPX3 expression was associated with less favorable prognosis in GBM, OV, LUSC, UCEC, ESCA, LIHC, STAD, READ, and SKCM (p<0.05) (**Figure 7D**). GPX3 High expression was associated with favorable prognosis in LUAD, PAAD, KIRP, LGG, and KICH (p<0.05) (**Figure 7D**). These results demonstrated that the expression of CALB2 and GPX3 was intimately related to the prognosis of various cancer types.

### Chemotherapy drug sensitivity

**Figure 8:**
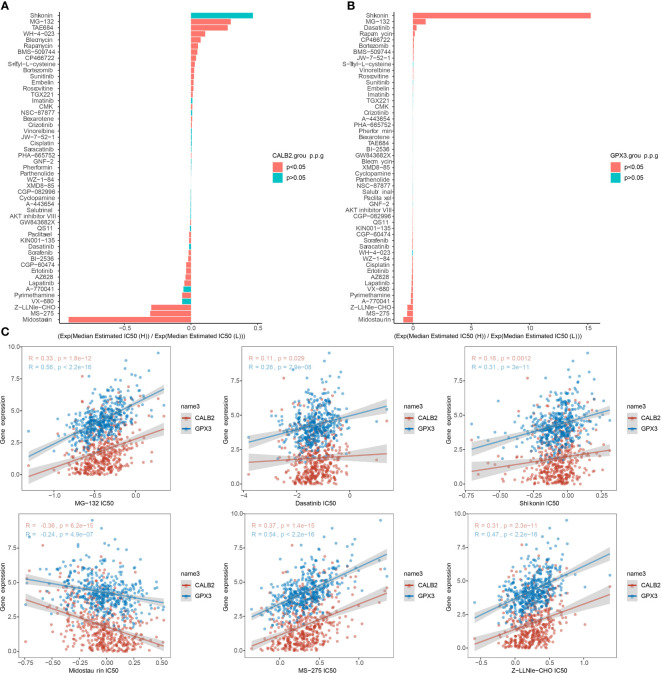
Chemotherapy drug sensitivity. **(A, B)** Histogram of CALB2 and GPX3 drug sensitivity analysis **(C)** Correlation of CALB2 and GPX3 with IC50 of chemotherapeutic agents.

We compared the IC50 data of 51 chemotherapeutic agents in the high and low expression groups of CALB2 and GPX3 (**Figures 8A, B**). The three groups with the largest and smallest IC50 values were selected for correlation analysis with CALB2 and GPX3 expression. We found that CALB2 and GPX3 expression were consistent with the drug response trend of MG-132, Dasatinib, Shikonin, Midostaurin, MS-275, and Z-LNle-CHO. The expressions of CALB2 and GPX3 were positively correlated with the IC50 of MG-132, Dasatinib, Shikonin, MS-275 and Z-LLNle-CHO. The expressions of CALB2 and GPX3 were significantly negatively correlated with the IC50 in Midostaurin (**Figure 8C**). These results suggested that GEM combined with MG-132, Dasatinib, Shikonin, Midostaurin, MS-275, and Z-LLNle-CHO might treat CRC through the action of CALB2 and GPX3.

## Discussion

CRC was characterized with high tumor heterogeneity, tumor mutation-resistant cells increasing the challenge of chemotherapy treatment, and new targets are crucial in CRC treatment (3). In this study, comprehensive bioinformatics analysis identified CALB2 and GPX3 which functioned as important targets in GEM treatment of CRC.

CALB2 encodes a Ca2+ binding protein that was intimately associated with cancer (22). a study by Bertschy et al. determined that CALB2 was expressed mainly in nervous system cells or ovarian cells (23). Further studies demonstrated that CALB2 was specifically expressed in CRC and mesotheliomas, which was considered as well as the diagnostic biomarker for CRC and mesotheliomas (23–27). In a recent study, Ojasalu et al. (28) demonstrated through *In-vitro* assays that CALB2 silencing inhibits ovarian high-grade plasmacytoma (HGSC) cell adhesion, which in turn caused peritoneal spread, and notably, that high CALB2 expression contributed to poor prognosis of HGSC.GEM was primarily subject to enzymatic deamination, low clearance, and drug resistance and It was currently intended primarily as alternative second-line therapeutic agent to 5-FU for the treatment of multiple cancers (4). At the cellular level, GEM is internalized *via* nucleic acid transporters. It is subsequently phosphorylated by dioxycytidine kinase (DCK). The stepwise phosphorylation leads to the formation of GEM-triphosphate, which is incorporated into cellular DNA, thereby inhibiting nuclear replication (7). Recent research concluded that GEM held promise as tumor-targeting agent by optimizing the mode of drug delivery action (7). Stevenson et al. (29) established that CALB2 in CRC responded to 5-FU regulation and that expression of down-regulated CALB2 induced death of CRC cells. Numerous investigations proved that CALB2 was the key gene in CRC development as well as treatment. Excitingly, the present report identified CALB2 as the possible hub gene affecting the potency of GEM through bioinformatics approaches. our pan-cancer analysis similarly established that CALB2 probably served as prognostic biomarker for multiple cancer species, thus illustrating that GEM-CALB2 might be promising for novel therapeutic modalities in cancer.

GPX3 transcription was regulated by selenium (5) and peroxisome proliferator-activated receptor γ (PPARγ), which protected cells against reactive oxygen species (ROS) accumulation (30–32). The finding in this study that GPX3 low expression caused poor prognosis in CRC was also demonstrated in earlier studies. The findings of Barrett et al. (33) found accelerated tumor accretion and significantly higher number of tumor cells in GPX3-deficient COAD mice, which also exhibited macrophage tendency to M2 polarization, enhanced expression of inflammatory factors, and over-activation of WNT signaling pathway.GPX3 in COAD mice exhibited immunomodulatory effects limiting the development of enteritis-associated cancers. Another investigation confirmed that downregulation of GPX3 expression led to increased H2O2 levels in TME and promoted tumor malignancy (34). Enrichment analysis in this study revealed that GPX3 was closely associated mainly with immune and inflammation-related pathways and that increased GPX3 expression could inhibit the metabolic response of the organism. Our study was consistent with the results of previous studies. In addition, Ji et al. (35) found that the administration of GEM induced ROS generation in HCC and activated Ets2 to upregulate CD13 expression, and the activated expression of CD13 induced GEM resistance by activating NRF1 to upregulate GPX3 expression to clear intracellular ROS levels in HCC. This showed that GPX3 was closely associated with GEM potency, which was further confirmed by our study.

For CRC treatment, combination drug treatment modalities were feasible strategies (36). A recent report suggested that the combination of drugs could appropriately prolong the survival of CRC patients compared to chemotherapy alone (2). There was no exact effective targeted therapy for patients with high variability (2). The evaluation indexes of drug sensitivity generally include Area Under the Curve (AUC), Half maximal inhibitory concentration (IC50), Half maximal effective concentration (EC50) and Maximal effect level (Amax) (37–39). But IC50 is by far the most used. Therefore, tapping the exact therapeutic target is an urgent issue for CRC treatment. In this study, CALB2 and GPX3 expression were found to be consistent with the drug response trends of MG-132, Dasatinib, Shikonin, Midostaurin, MS-275, and Z-LNle-CHO, and CALB2 and GPX3 were potential pharmacodynamic targets of GEM. We hypothesized that the combination of GEM with MG-132, Dasatinib, Shikonin, Midostaurin, MS-275, and Z-LLNle-CHO may target CALB2 or GPX3 for CRC. These results demonstrated that CALB2 and GPX3 might be hub genes for GEM action.

Although this investigation integrated several databases to explore the hub genes affecting the potency of GEM, there were still shortcomings in this study. First, the integrated bioinformatics results provided that CALB2 and GPX3 were possible hub genes for GEM action, but there was no *In-vitro* cellular assay or *in-vivo* assay to validate this result, and subsequent wet experiments needed to be designed to further validate our results. Second, we determined that CALB2 and GPX3 enhanced immune function in CRC patients, but we did not conduct in-depth studies to explore the molecular mechanisms involved. Subsequent studies will focus on the specific regulatory mechanisms of GEM on CALB2 and GPX3 as well as a large sample multicenter prospective study to explore the effects of GEM combination with targeted therapies on CRC, leading to the development of novel therapeutic tools. Overall, this study revealed that CALB2 and GPX3 are potential target genes for GEM action.

## Conclusion

CALB2 and GPX3 served as biomarkers of CRC prognosis and as potential target genes for GEM. Our study provided new thought for the development of novel combination drug-targeted therapies for CRC.

## Data availability statement

The original contributions presented in the study are included in the article/**Supplementary Material**. Further inquiries can be directed to the corresponding authors.

## Author contributions

All authors contributed to this present work. XXZ and LL designed the study, LS acquired the data. XL and YJ drafted the manuscript, JS and XHZ revised the manuscript. All authors read and approved the manuscript.

## Glossary

**Table:**

CRC	colorectal cancer
GEM	Gemcitabine
TIIC	tumor-infiltrating immune cells
TME	tumor microenvironment
TCGA	The Cancer Genome Atlas
GEO	GENE EXPRESSION OMNIBUS
IC50	half maximal inhibitory concentration
GDSC	Genomics of Drug Sensitibity in Cancer
WGCNA	Weighted correlation network analysis
PCA	principal component analysis
CDRGs	CRC development-related genes
K-M	Kaplan-Meier
GSVA	Gene Set Variation Analysis
ESTIMATE	Estimation of STromal and Immune cells in MAlignant Tumours using Expression data
HGSC	high-grade plasmacytoma
5-FU	5-fluorouracil
PPARγ	peroxisome proliferator-activated receptor γ
ROS	reactive oxygen species.
