# Supplementary material for: Comprehensive analysis identifies novel targets of gemcitabine to improve chemotherapy treatment strategies for colorectal cancer
Source: Front Endocrinol (Lausanne). 2023 Aug 17;14:1170526. doi: 10.3389/fendo.2023.1170526 (PMC10471186; doi:10.3389/fendo.2023.1170526)
Supplement: Supplementary file 1 [file DataSheet_1.pdf]

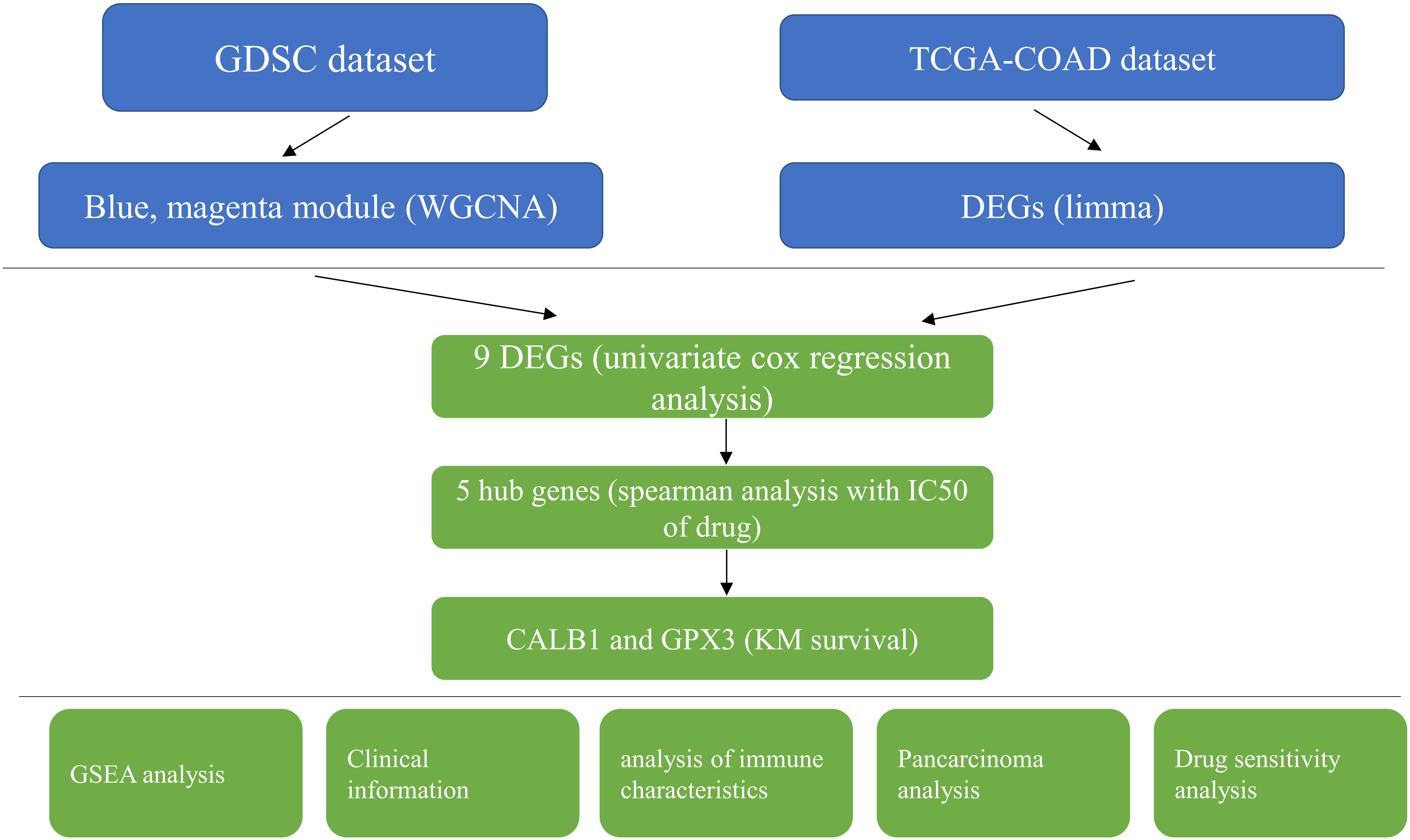
GDSC dataset
TCGA-COAD dataset
Blue, magenta module (WGCNA)
DEGs (limma)
9 DEGs (univariate cox regression analysis)
5 hub genes (spearman analysis with IC50 of drug)
CALB1 and GPX3 (KM survival)
GSEA analysis
Clinical information
analysis of immune characteristics
Pancarcinoma analysis
Drug sensitivity analysis
