# Supplementary figures and images for: Comprehensive analysis identifies novel targets of gemcitabine to improve chemotherapy treatment strategies for colorectal cancer
Source: Front Endocrinol (Lausanne). 2023 Aug 17;14:1170526. doi: 10.3389/fendo.2023.1170526 (PMC10471186; doi:10.3389/fendo.2023.1170526)

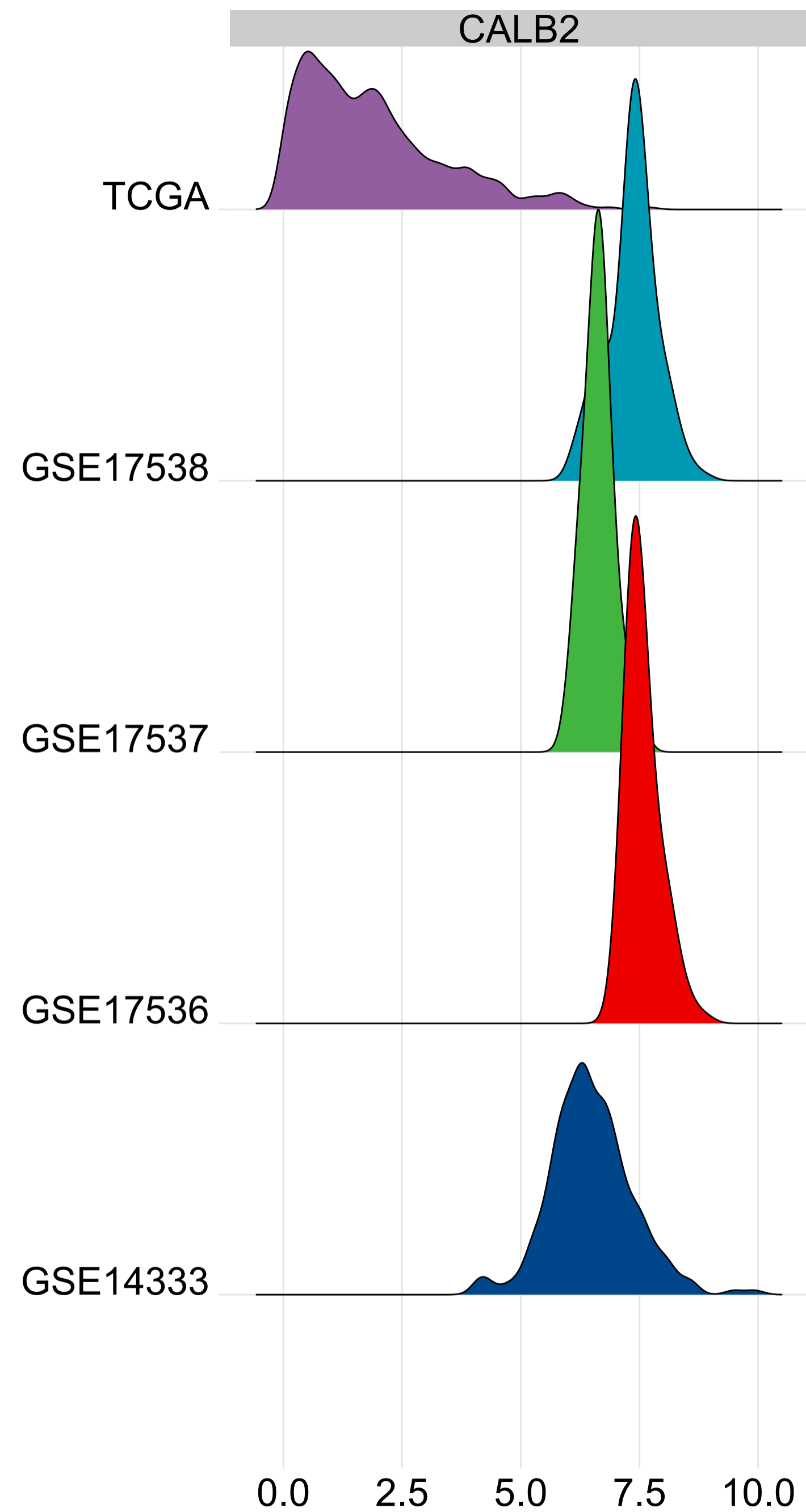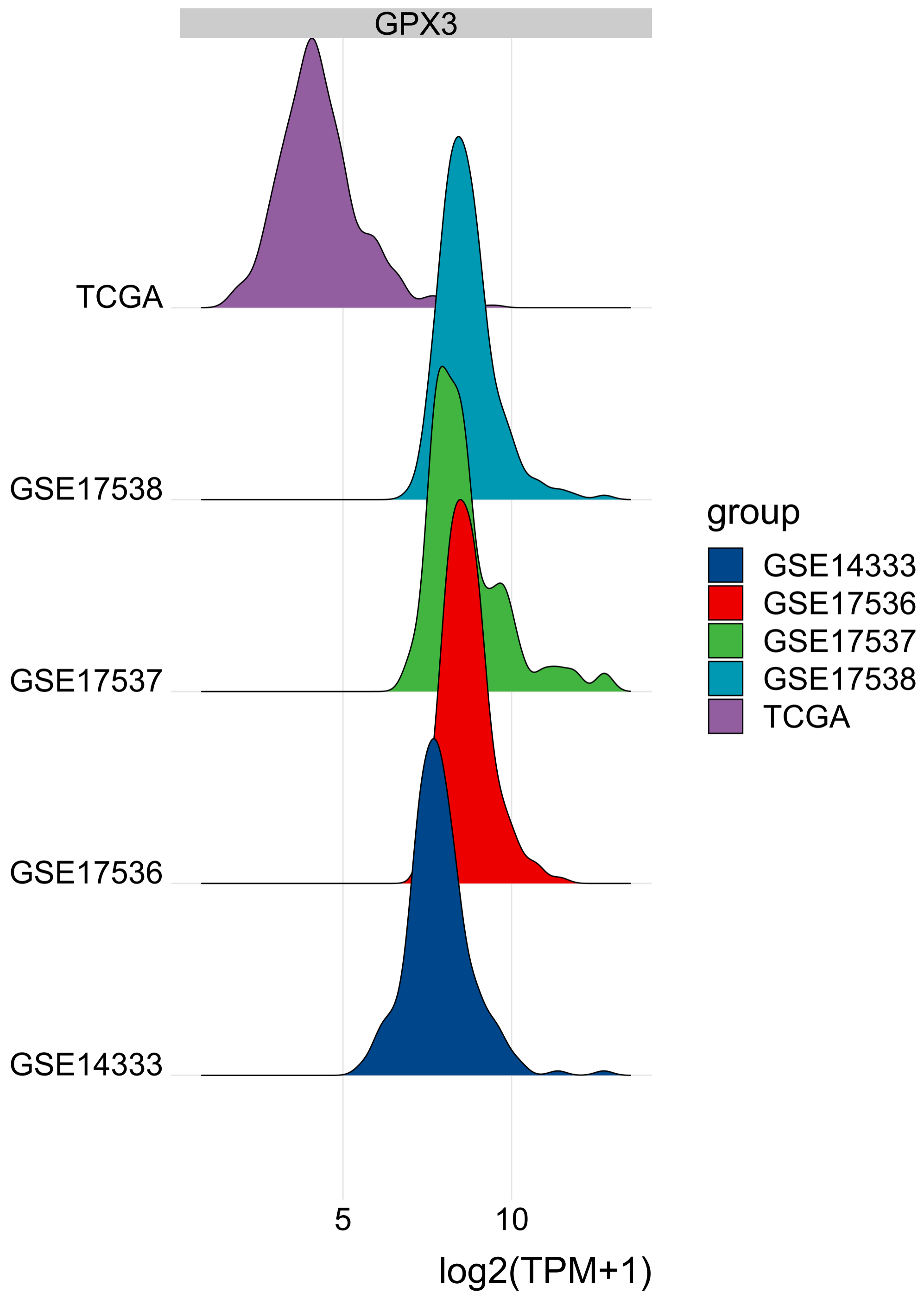

Supplement: Supplementary file 2 [file DataSheet_2.pdf]

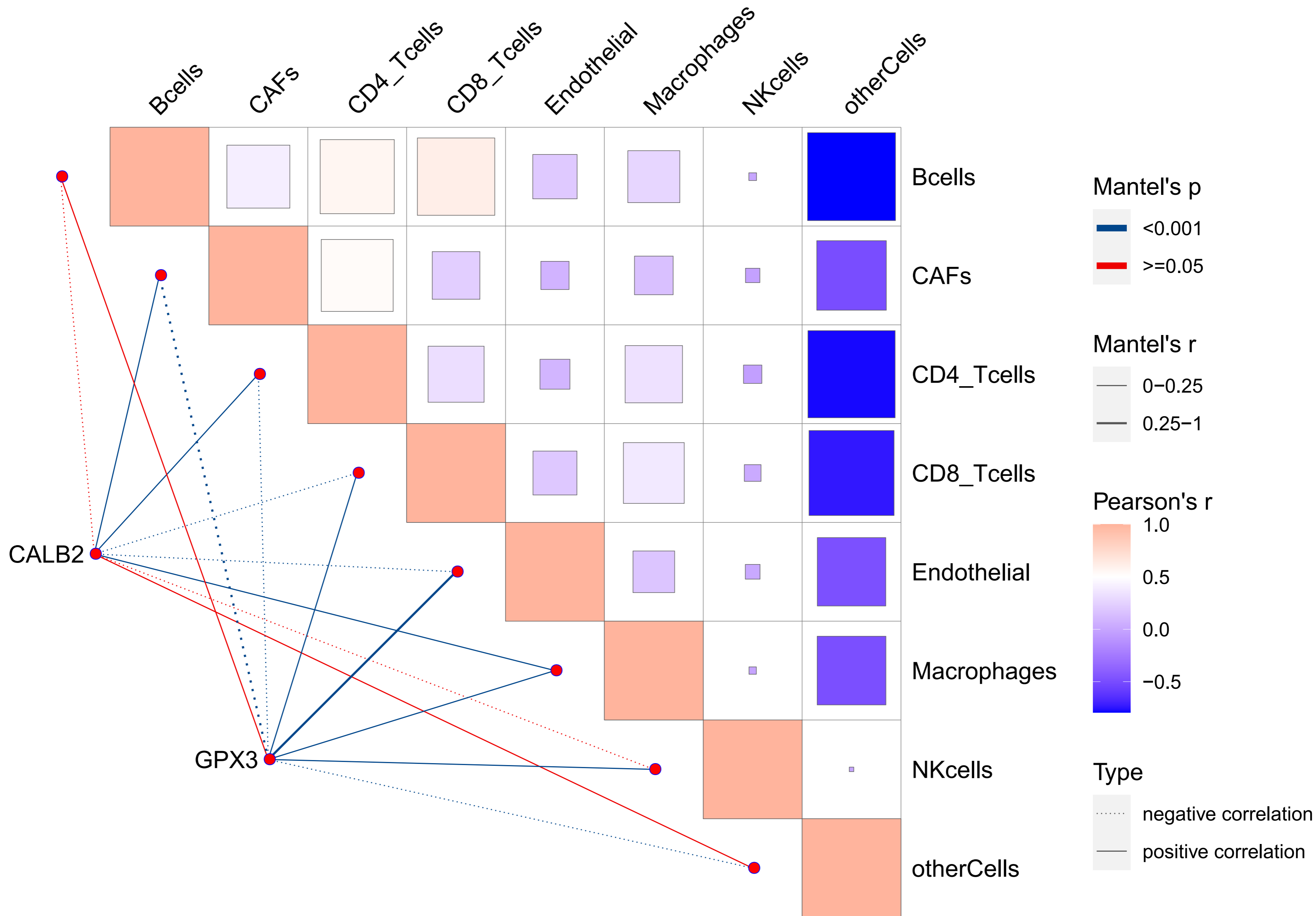

Supplement: Supplementary file 3 [file DataSheet_3.pdf]
